# MENTAL AND PHYSICAL HEALTH CONDITIONS LIMITING ACTIVITIES IN A NATIONAL SAMPLE OF OLDER LGBT ADULTS

**DOI:** 10.1093/geroni/igae098.0111

**Published:** 2024-12-31

**Authors:** Ilan Meyer, Lauren Bouton, Diane Lauderdale

**Affiliations:** University of California Los Angeles, Los Angeles, California, United States; University of California Los Angeles School of Law, Los Angeles, California, United States; Public Health Sciences University of Chicago, Chicago, Illinois, United States

## Abstract

AIMS: Research suggests that the proportion of LGBT older adults experiencing disabilities and limitations in activities may be high compared with cisgender straight older adults, but we know little about these limitations. We aimed to understand the type and reasons for limitations in LGBT older adults. METHODS: Using a national probability sample of 210 LGBT adults 45 years and older we conducted in-person or zoom interviews and asked respondents if they were “limited in activities because of physical, mental, or emotional problems?” We followed with an open-ended question about the reason for endorsing having limitations. RESULTS: 36% of respondents reported 1 or more limitations. We categorized these limitations as related to mental health (17%), mobility (72%), pulmonary conditions (7%), sensory/cognition (8%), or cardiovascular including diabetes and obesity (8%). Overall limitations were more prevalent among respondents 65 years or older vs. 45-64 years old (47% vs. 30%), women vs. men (48% vs. 28%), White vs. POC (38% vs. 31%), bisexual and other sexual identities vs. LG (50% vs. 31%), and people with incomes below $49K vs. $50k or higher (48% vs. 32%). Reporting a chronic disease diagnosis (e.g., asthma, hypertension) vs. none was not associated with prevalence of limitations (37% vs. 36%) but self-rated poor/fair health was (58% vs. 31%). In age-adjusted analyses, being a woman, identifying as other than LG, lower income, and reporting poor/fair health were all associated with having limitations (RRR = 2.7, 3.3, 2.1, and 3.5, respectively). We will discuss predictors of observed high prevalence of limitations.

